# Determinants of timely initiation of complementary feeding among children aged 6–23 months in Ethiopia

**DOI:** 10.1038/s41598-022-21992-w

**Published:** 2022-11-09

**Authors:** Girma Gilano, Sewunet Sako, Kasarto Gilano

**Affiliations:** 1 Department of Health Informatics, School of Public Health, College of Medicine and Health Sciences, Arba Minch University, Arba Minch, Ethiopia; 2 Department of Public Health, Arba Minch College of Health Sciences, Arba Minch, Ethiopia

**Keywords:** Health care, Medical research

## Abstract

Introducing complementary feeding either early or later than 6 months is associated with future negative health outcomes. However, many women in Ethiopia do not follow WHO standard time to feed their children, which might be due to various demographic, economic, access, and availability of services. Thus, we aimed to identify factors attributing to the problems to assist future interventions. We used cross-sectional EMDHS 2019 for this analysis. We cleaned the data and 4061 women with under 2 years children were identified. We applied multilevel binary logistic regression in Stata v.15. Model comparison was based on log-likelihood ratio, deviance, and other criteria. We presented data using mean, percent, 95% CI, and adjusted odds ratio (AOR). The timely complementary feeding was 36.44% (34.93–37.92%). Factors like preceding birth intervals (AOR = 1.97 95% CI 1.62–1.39), primary education (AOR = 2.26 95% CI 1.40–3.62), secondary above education (AOR = 1.62 95% CI 1.10–2.38), and rich wealth index (AOR = 1.25 95% CI 1.03–1.52) were some of the associated factors. The magnitude of timely initiation of complementary feeding was diminutive. Authors suggest that interventions considering maternal education, empowering mothers economically, equity access to health services, and birth planning a good remedy.

## Introduction

In the 2019 Ethiopian Mini Demographic and Health Survey (EMDHS) report and World Health Organization (WHO) child feeding recommendation manual, complementary feeding is defined as feeding a child with solid and liquid foods in addition to breast milk^1^. Similarly, complementary feeding introduced at the age of 6th to 8th months is timely and outside this time range is untimely complementary feeding^1–3^. According to the WHO, exclusive breastfeeding is no-more enough to support child growth and development at and beyond the sixth month. Thus, children need breast milk plus additional (complementary) feeding to fill the lagging nutrients gap that is known for 45% of child deaths in low and middle-income countries^3,4^. Some studies defined timely complementary feeding as the food combined with breast milk within the 6–8 months after birth^5–7^. The United Nations Children’s Fund (UNICEF) informs that families have been challenged by economic, political, market, social, or cultural barriers to feed their children affordably and safely in every corner of the world. Additionally, inappropriate complementary feeding affects 149 million children around the globe^4,8^. In addition, the magnitude of the problem is relatively higher in Sub-Saharan countries.

In South Asian countries, the untimely initiation of complementary feeding ranged from 17 to 76% in Bangladesh^6^. It was 61% in Pakistan^5^ followed by 43.6% in Nepal^9^. In Sub-Saharan countries, the average untimely initiation of complementary feeding is 31.7% in 2019^10^. Similarly, a systematic review and meta-analysis in the Sub-Saharan region showed that 44.19% of mothers do not start complementary feeding as per WHO recommended time^11^. This is further explained by 62.5% of mothers initiating complementary feeding within 3-5 months in Nigeria^12^. In Ethiopia, a study in the Maichew district showed that around 40% of the mothers do not know the exact time of initiating complementary feeding^13^. And another study in Addis Ababa showed that 17% of mothers began complementary feeding earlier than 6 months ^14^. Another study in Dessie showed that 13.1% and 21.8% of mothers started giving complementary feeding earlier and later than recommended time respectively^15^. Additionally, in the Northwest Ethiopia, 47.2% of mothers also practiced untimely complementary feeding^16^, and it is 37% in the Northeast Ethiopia^17^. The new evidence from a systematic review and meta-analysis in 2020 showed that 34.4% of the mother do not start complementary feeding at the recommended time^18^.

Previous studies already identified some of the predictors of untimely complementary feeding. In South Asia, lack of complementary feeding knowledge, low maternal education, socio-economic status, and cultural beliefs were the factors attributable to untimely complementary feeding^5^. Family iincome, lack of knowledge, and incorrect advice were some of the factors mentioned in another study^19^. A study conducted in Nigeria showed that orthodox maternity care, exclusive breastfeeding, and absence of the siblings were associated with the timely initiation of complementary feeding^20^. In Ethiopia, women’s employment, husband education, birth preparedness, growth monitoring, knowledge of time to introduce complementary feeding, and parental support were some of the factors affecting the time to initiate complementary feeding among mothers^7,13,21–23^. In another study, maternal education, complementary feeding counseling, and maternal knowledge also influenced the timing of initiating complementary feeding^15^. From this section, we understand that there is international, national, and regional shreds of evidence related to the topic area. However, the evidence is inconsistent throughout and inconclusive to trigger coordinated interventions. It indicates that further studies are necessary to find out critical factors to trigger intervention. In addition, there was no large-scale study addressing this topic since 2016. It is also important to assess if access, availability, socioeconomic, and socio-demographic changed over the last 5 years and if different decisions are necessary at country level to improvement the magnitude of problem.

Additionally, studies shows that a remarkable number of mothers do not adhere to the World Health Organization (WHO) complementary feeding recommendations; however, there is limited information on the larger population (country-level samples) for policy and decision-makers currently. Thus, this study had the aim of identifying factors enforcing mothers for untimely complementary feeding to provide the most recent representative information for further policy decisions from the recent country-level data using multilevel logistic regression that accounts for regional differences.

## Methods

### Study setting and data source

Ethiopia has conducted two EMDHS recently. In the 2019 EMDHS, the data collection was a community-based cross-sectional carried out from March 21, 2019, to June 28, 2019. All the nine regional states of the country (Afar, Tigray, Amhara, Oromia, Somali, Southern Nations, Nationalities, and People’s Region (SNNPR), Benishangul Gumuz, Gambella, and Harari) and two city administrations (Addis Ababa and Dire Dawa) were included in the study.

We used secondary data (EMDHS) for this analysis. EMDHS used a stratified two-stage cluster sampling. Randomly, the Enumeration Areas (EA) have selected in the first stage and then households have selected in the second stage. In all selected households, height, weight measurements, and all nutritional data were collected from children aged 0–59 months. We included 4,061 women who are 15–49 year old from face-to-face interview questinnaire^24^. The details of the recorded data is now available from the measure program web address: (http://www.dhsprogram.com.). We extracted 4,061-weighted data from mother and children who live with their mothers for this analysis.

### Study variables

#### Outcome variable

The outcome of this study was the timely initiation of complementary feeding. Complementary feeding is defined as the time when feeding other than breast milk is initiated between 6 and 8-months. It is untimely when it is commenced before 6 months or beyond 8 months. Below 6 and above 8 month initiation complementary feeding recoded as ‘0’ (no/not timely), and 6–8 month evidence of initiating complementary feeding recoded as ‘1’ (yes/timely).

#### Independent variables

The explanatory variables are the socio-demographic of the family, maternal services, and nutritional factors (Table 1).

**Table 1:** Coding and description of explanatory variables.

No	Variable	Description	Code
1	Region^*^	The 11 regional location of the households included in the study	1 = Tigray, 2 = Afar, 3 = Amhara, 4 = Oromia, 5 = Somali, 6 = Benishangul-Gumuz, 7 = SNNP 8 = Gambela, 9 = Harari 10 = Addis Ababa, 11 = Dire Dawa
2	Place of residence^*^	Type of place of residence	1 = Urban, 2 = Rural
3	Less than 5 yr children	No of children < 5 yr in household	1 = 0-1child, 2 = 2 children, 3 = ≥ 3children
4	Mother’s Education	Mother’s level of education achieved	0 = No education 1 = primary2 = secondary 3 = Higher and above
5	Place of delivery	Place of delivery	0 = Home, 1 = Health Sector
6	Breastfeeding	Breast feeding status	0 = not breastfed, 1 = exclusive, 2 = breastfed + plain water, 3 = breastfed + non-milk liquid, 4 = breastfed + complementary food
7	Wealth index	Wealth index of household	0 = Poor, 1 = Medium 2 = Rich
8	Sex	Sex of child	1 = Male,2 = Female
9	Birth order	Birth order of child	1 = first order, 2 = 2^nd^, 3 = 3^rd^ or greater
10	Current age	Current age of mother	0 = 15-24 years, 1 = 25-34 years, 2 = 35-49 years
11	birth interval	Preceding birth interval (months)	0 = ≤ 24 months, 1 = 25-35 months, 2 = ≥ 36 months
12	Household head	Head of house hold	1 = Male, 2 = Female
13	Vitamin A	Child received vitamin A	0 = No 1 = Yes
14	Contraception	Current use by method	0 = Traditional 1 = Modern

*community-level factors.

#### Data processing and analysis

We used frequencies, weighted frequencies, mean, standard deviations, and percentages or proportions to describe the timely initiation of complementary feeding. We also calculated the mean Variance Inflation Factor (VIF = 1.23) is in the acceptable range. We also applied sampling weight to manage the representativeness of the survey and to account for sampling design when calculating standard errors.

We used a multilevel mixed-effects logistic regression model to analyze the data since EMDHS data has some structures. The data violates the independency of observations and the equal variance assumption of the traditional logistic regression model. In the current model, we fitted four models to estimate both fixed and random effects variables. We used the null model (intercept only model) to check the magnitude of intra-cluster variability. Second, we included all individual-level factors in the model (Model I). Additionally, Model II was fitted with community-level variables. And finally, we combined individual-level (fixed effect) and community-level (random effect) factors to form a mixed effect model (Model III) to identify factors associated with timely initiation of complementary feeding. Intra-cluster correlation is ICC $= \frac{\partial 1}{{\partial 1 + \pi^{2/3)} }}$; where, $\partial 1$ is the variance of the null model and $\pi^{2/3}$ is the fixed number 3.29. Proportional Change in Variance is PCV $= \frac{\partial 1 - \partial n}{{\partial 1}}$; where $\partial 1$ is the null model variance and $\partial n$ is thevariance of the neighborhood in the subsequent model. Median Odds Ratio is MOR $= {\text{e}}^{{0.95\sqrt {\partial 1} }} { }$; where, $\partial n$ is the variance of the null model. We used log-likelihood, deviance, AIC, and BIC to compare models and identified the best-fitted model using. We checked each variable for significance at *p* < 0.20 to include in models and used *p* < 0.05 for the final association indication. We cleaned the data as per the study criteria and analyzed it in STATA v. 15.0 after weighting.

### Ethical approval and consent to participate

Ethical clearance for the 2019 EMDHS was provided by the Ministry of Health ethics committee, the National Research Ethics Review Committee (NRERC), the Institutional Review Board of Inner City Fund (ICF) at DHS program internationally, and the Government of Ethiopia. The author obtained the 2019EMDHS data in different reading formats by online request at the DHS program. The authors also confirm that all methods were carried out in accordance with relevant guidelines and regulations.

## Results

We analyzed 4061 children’s initiation of complementary feeding time and found 36.44% (34.93–37.92%), meaning more than 63% of families in Ethiopia initiate their children complementary feeding either earlier or later than the recommended time. Participants from agrarian regions accounted for 87.81% of this proportion. Nearly sixty percent (59.69%) of the mother were aged 25–34. Additionally, 50.10% of the mothers had two children under 5 years old, 61.98% of mothers were not learned, 48.85% of mothers were from poor wealth index families, 21.69% of preceding intervals were below 24 months, and 58.32% of mothers gave birth at their home (Table 2).

**Table 2:** Factors associated with timely initiation of complementary feeding in Ethiopia, EMDHS 2019.

Variable	Unweighted (%)	Weighted (%)	Variable	Unweighted (%)	Weighted (%)
**Regions**			**Age of mother**		
Agrarian	2762 (66.22)	3566.53 (87.81)	15–24	572 (13.71)	570.20 (11.58)
Pastoralist	992 (23.78)	387.76 (9.55)	25–34	2514 (60.27)	2424.31 (59.69)
City administrations	417 (10.00)	107.12 (2.64)	≥ 35	1085 (26.01)	1166.90 (28.73)
**Number of children < 5 yr**			**Mother’s Education**		
1	1276 (30.59)	1351.96 (33.29)	No education	2605 (62.46)	2517.12 (61.98)
2	2045 (49.03)	2034.90 (50.10)	Primary	1151 (27.60)	1233.25 (30.37)
≥ 3	850 (20.38)	674.55 (16.61)	Secondary & above	415 (9.95)	311.04 (7.66)
**Birth interval**			**Wealth index**		
≤ 24 months	966 (23.16)	880.81 (21.69)	Poor	2282 (54.71)	1984.01 (48.85)
25–35 months	1233 (29.56)	1135.64 (27.96)	Middle	595 (14.27)	760.37 (18.72)
≥ 36 months	1972 (47.28)	2044.90 (50.35)	Rich	1294 (31.02)	1317 (32.43)
**Breast feeding status**			**Place of delivery**		
Not breastfed	2442(58.55)	2264.93(55.77)	Home	2329 (55.84)	2368.43 (58.32)
Exclusive breastfed	840 (20.14)	926.82 (22.82)	Health facility	1842 (44.16)	1692.98 (41.68)
Breastfed + other liquids	98 (2.35)	57.67 (1.42)	**Child received vitamin A**		
Breastfed + complementary food	791 (18.96)	811.99 (19.99)	No	1358 (55.27)	1320.96 (55.82)
			Yes	1099 (44.73)	1045.32 (44.18)

The analysis of factors associated with timely initiation of complementary feeding showed that the maternal age, maternal education, preceding birth interval, number of under five children, sex of the household leader, and the wealth index were significant. City administration City administration is the only significant random effect variable. Except for the sex of the household leader, all those variables were also significant under the final model (mixed effect model). We interpreted variables from the last model below. Accordingly, mothers with the age range of the 25–34 and the age ≥ 35 years had 40% and 53% reduced odds of untimely complementary feeding with AOR of 0.60 (0.49–0.74) and 0.47 (0.37–0.60) respectively compared to age 15–24 years. Conversely, mothers who reported preceding birth intervals greater than 36 months had 1.97 times more like to start complementary feeding timely with AOR of 1.97 (1.62–1.39) compared to 24 months. Mothers who had two and more under-five children during the survey had higher odds of starting complementary feeding timely with AOR of 3.63 (3.03–4.36) and 4.12 (3.25–5.21) respectively. Primary and secondary and above maternal education is positively associated with higher odds of timely initiation of complementary feeding with AOR of 2.26 (1.40–3.62) and 1.62 (1.10–2.38) respectively. Respondents from the rich family wealth index had high odds of reporting timely complementary feeding with AOR of 1.25 (1.03–1.52). At the community level, respondents from pastoralist regions had 33% reduced odds of initiating timely complementary feeding with AOR of 0.77 (0.61–0.98), and those from city administrations had higher odds of reporting timely complementary feeding with AOR of 1.47 (1.11–1.96) (Table 3).

**Table 3:** Multilevel analysis of timely initiation of complementary feeding among aged 6–23 months in Ethiopia, 2019 EMDHS.

Variables	Model 0	Model I	Model II	Model III
**Age in 5 years group**			–	
15–24	–	1	–	1
25–34	–	0.62 (0.51–0.76)***	–	0.60 (0.49–0.74) )***
≥ 35	–	0.49 (0.38–0.62)***	–	0.47 (0.37–0.60) )***
**Education**	–		–	
No education	–	1	–	1
Primary education	–	2.39 (1.49–3.82)***	–	2.26 (1.40–3.62)***
Secondary education & above	–	1.78 (1.22–2.60)**	–	1.62 (1.10–2.38)**
**Preceding birth interval**	–		–	
< 24 months		1		–
24-36 months		1.04 (0.86–1.25)		1.03 (0.85–1.24)
> 36 months		2.00 (1.65–2.43)***		1.97 (1.62–1.39)***
**Number of children < 5 yr**	–		–	
1	1			
2		3.63 (0.03–4.35)***		3.63 (3.03–4.36)***
≥ 3		3.99 (3.16–5.04)***		4.12 (3.25–5.21)***
**Sex of household leader**	–		–	
Male	–	1		
Female	–	1.17 (1.02–1.47)*		1.10 (1.07–1.68)
**Wealth index**	–		-	
Poor		1		
Middle		1.02 (0.81–1.25)		0.99 (0.79–1.23)
Rich		1.25 (1.08–1.67)**		1.25 (1.03–1.52)*
**Region**	–			
Agrarian		–	1	1
Pastoralist			0.91 (0.73–1.12)	0.77 (0.61–0.98)*
City administrations			1.52 (1.13–2.04)**	1.47 (1.11–1.96)**

NB: * = *p* < 0.05; ** = *p* < 0.01; & *** = *p* < 0.001.

Although the data is not highly affected by clusters, the results from Table 4 shown, the model fitting with balancing the existing hierarchies is adequate. The decreased ICC, AIC, BIC, deviance, and the increased log-likelihood ratio showed how the model improved over the multiple modeling. The 2% unaccounted ICC can be raid off by including additional random effect variables (Table 4).

**Table 4:** Model comparison and random effect distribution of timely initiation of complementary feeding among children of 6–23 months age in Ethiopia, 2019 EMDHS.

Random effect model comparison	Model 0	Model 1	Model 2	Model 3
Community-level Variance	0.25	0.15	0.10	0.08
Inter-cluster correlation (ICC)	0.066	0.04	0.03	0.02
Log likelihood ratio (LLR)	− 2729	− 2572	− 2722	− 2566
Proportional change in variance (PCV)	Ref	0.4	0.6	0.68
Media odds ratio (MOR)	1.60			
AIC	5463	5171	5455	5163
BIC	5476	5254	5487	5258

## Discussion

From our analysis, only 36.44% (95% CI 34.93–37.92%) of children started their complementary feeding within the WHO recommended time, which means nearly 64% of the children began complementary feeding either before 6 months or later than 8 months. The 64% untimely complementary feeding magnitude is less than 76% in Bangladesh^6^, but consistent with 61% in Pakistan^5^, and 62.5% in Nigeria^12^. It is greater than 43.6% in Nepal^9^, 44.19% in the Sub-Saharan region^11^, 47.2% in Northwest Ethiopia^16^, 37% in Northeast Ethiopia^17^; and 34.4% pooled prevalence in Ethiopia^18^. This means the finding is greater than the South Asian, regional, and country-level average untimely proportions. The higher untimely initiation of complementary feeding might show that mothers were more engaged in formula feeding as modified products are available and families might be exposed to a promoted product that might trigger usage at an inappropriate time. Some developing countries are worse than Ethiopia where untimely complementary feeding is higher and this might indicate the negative social, economic, and media (promotion) factors contribute higher in those countries. Additionally, 50.10% of the mothers had two children aged below 5 years. One study showed that most mothers are young and had ≥ 7 children^25^. This might be due to the shorter birth interval practiced by women in Ethiopia. It is not a secret that a 27.96% birth interval is around 35 months, which means mothers have the possibility of giving birth to another child before the fifth birthday of the preceding child. In other words, 61.98% of the mother had no education. This is supported by 62.8% of poor education in Nigeria^20^ and 54.0% in North Ethiopia but different from 30% in Northwest Ethiopia^17^. The consistency might indicate the poor achievement in maternal education both regionally and at country-level disquiet the situation. Maternal education is good in some small-scale studies that might show uneven distribution of maternal education in the country. In addition to this, 48.85% of mothers were from poor-wealth index families. This is also supported by many studies^11,15,17,26^. It might indicate that poor families are practicing more untimely complementary feeding. Poor families might be poorly educated (mothers) and income limited families where breast milk is not enough for their children and therefore start complementary feeding outside the recommended time. Overall, the economic status of people in the country is poor, so supporting mothers economically and promoting women’s education could be worth a lot.

During multilevel modeling, age of the mothers, maternal education, preceding birth interval, the number of children under 5 years old per woman, and wealth index—the fixed-effect factors, while pastoralists and city administrations—the random effect factors were significant. Mothers aged greater than 24 years were inversely associated with the timely initiation of complementary feeding in Ethiopia. Another study supported this idea^23^; however, mothers of age < 20 years were associated with the timely complementary feeding compared to the higher groups^27^. This might show that as age increase number of children increase and maternal breastfeeding capacity decrease and trigger untimely complementary feeding. Maternal education is an independent predictor of the timely initiation of complementary feeding. Many previous studies supported this concept in the country^5,6,9,15,17,20^. It means maternal education is an appealing intervention that undivided attention. As the preceding birth interval increase above 36 months, the probability of mothers sticking to the recommended time of initiating complementary feeding increases. Some studies support this in the country^19,21^. It might means child spacing special courtesy. However, it might also mean those mothers who were with good education, use family planning, and with good economic support only receives the service. Mothers with a rich wealth index had good timely initiation of complementary feeding, the impression also supported by other studies^15,17^. The consistency might be because rich mothers can buy baby formula and start complementary feeding untimely. It might also mean rich mothers are ignoring standard recommendations. Mothers from pastoralist regions do not practice timely complementary feeding while, mothers from city administrations did well. The regional difference regarding complementary feeding is also immense in another study^28^. The difference might be due to differences in equity distribution of health services, access, availability, and maternal education-related matters. Despite these finding of this study, some limitations need considerations. Disproportion of sampling, high missing in the data, secondary nature of the data, and others were some of the problems which authors approached through weighting, reducing sample by missing, and considering the time of data collection in the discussion were involved.

## Conclusion

Depending on the finding of this study, the age of the mothers, maternal education, preceding birth interval, the number of children under 5 years old, wealth index, pastoralists, and city administrations need further commitment to have mothers practice timely complementary feeding. In addition to maternal education, wealth index and shorter birth intervals contributed to poor timing of complementary feeding. The availability of formula feeding for healthy children and the promotion of such products might be attracting many families to begin early complementary feeding. It might be difficult to tackle through the single best alternative. Therefore, governments, supplying (promoting) organizations, international and national organizations, and other stakeholders should work together for cumulative effect. Authors suggest critical attention is necessary to increase maternal education, women empowerment economically, access equity to service, and birth planning.

## Data Availability

The survey dataset used in this analysis is the third party data from the demographic and health survey website (www.dhsprogram.com) and permission to access the data is granted only for registered DHS data user.

## Abbreviations

DHS: Ethiopian Demographic Health Survey
EMDHS: Ethiopian Mini Demographic Health Survey
WHO: World Health Organization
SNNPR: South Nations Nationalities and Peoples Region
CI: Confidence Interval
AOR: Adjusted Odds Ratio
LL: Log Likely-hood
AIC: Akaike Information Criterion
BIC: Baye’s Information Criterion
UNICEF: United Nation Children’s Fund
ICC: Intra-Cluster Correlation
